# Compound Tincture of Iodoform

**Published:** 1879-09

**Authors:** 


COMPOUND TINCTURE OF IODOFORM.—TAKE of

| | |
|---|---|
| Iodoform - - - - | 15 grains |
| Potass, iodide - - - - | 2 drachms. |
| Glycerine - - - - - | 2 " |
| Strong alcohol - - - - | 6 " |

Rub up the iodoform and iodide of potassium, then add the glycerine, and rub it up to the consistence of thin cream, then add the alcohol and stir briskly. Dose, fifteen drops three times a day on sugar or in syrup.—*Canada Journal of Medical Science.*

